# Molecular imaging of atherosclerotic plaque targeted to oxidized LDL receptor LOX-1 using magnetic resonance

**DOI:** 10.1186/1532-429X-11-S1-O76

**Published:** 2009-01-28

**Authors:** Dayuan Li, Amit R Patel, Alexander Klibanov, Christopher M Kramer, Rene J Roy, Mirta Ruiz, David K Glover, George A Beller, Craig H Meyer

**Affiliations:** University of Virginia, Charlottesville, VA USA

**Keywords:** Atherosclerotic Plaque, Atherosclerotic Lesion, Aortic Wall, Total Imaging Time, Black Blood Imaging

## Background and objectives

Oxidized low-density lipoprotein and its receptor LOX-1 play a crucial role in the initiation, progression, and destabilization of atherosclerotic lesions. A noninvasive tool to improve the clinical characterization of this pathological process is needed. The aim of this study was to assess the feasibility of CMR based molecular imaging targeted to LOX-1 which is highly expressed on atherosclerotic lesions in mice.

## Materials and methods

LDLR-/- mice on an atherogenic diet for > 16 weeks were used. The imaging probe consisted of liposomes decorated with anti-LOX-1 antibody (or nonspecific IgG), gadolinium and DiI fluorescence markers. MRI at 7.0 T (Clinscan, Bruker/Siemens) was performed at baseline and 24 hrs after intravenous injection of 150 μl of probe containing LOX-1 antibody (n = 7) or nonspecific IgG (nIgG) (n = 5) with 0.075 mmol Gd/kg, followed by excision of the aorta for frozen cross-sections. The fluorescence image used to indicate whether the probe bound to the plaque was examined under fluorescence microscopy.

MRI of the ascending aorta was performed with a T1-weighted black-blood spiral gradient-echo sequence (echo time, 1.2 ms; flip angle, 90°; field of view, 3 × 3 cm; 135 interleaves; readout window, 4.1 ms; spatial resolution, 67 μm)) with 11 contiguous 0.5 mm-thick slices. Four signal averages with cardiac and respiratory gating were used, for a total imaging time of 2.5 minutes per slice. For the post-injection scan, the slices were matched to the baseline preinjection scan by using the left main and LAD coronary artery as anatomic landmarks.

To quantitatively analyze the MRI results, signal intensity (SI) was measured in 4 regions of interest within the aortic wall as well as the aortic lumen and muscle on each slice at both time points. The standard deviation of noise was also recorded for each slice. These measurements were recorded for all slices at every time point imaged. The contrast-to-noise ratio (CNR) of aortic wall to lumen was calculated for each slice. %CNR = (CNRpost-CNRprecontrast)/CNRprecontrast. The normalized enhancement ratio (NER) was defined as the average post-contrast SI from 4 regions of interest within the aortic wall divided by the muscle SI in the same slice and then divided by the pre-contrast SI. %NER = (NER-1) × 100.

## Results

**Figure 1:**
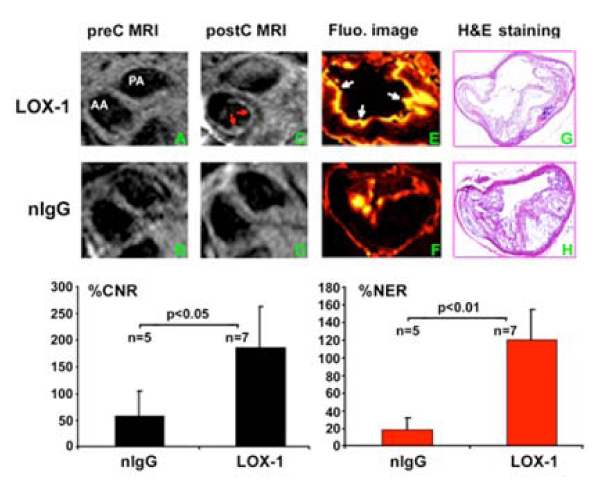
Figure 1

Fluorescence imaging found that the LDLR-/- mice injected with the LOX-1 antibody probe showed significant uptake in atherosclerotic plaque (Fig. 1E, white arrows). There was little fluorescence signal in atherosclerotic plaques in LDLR-/- mice that received the nIgG probe (Fig. 1F). The MR images consistently showed strong post-contrast signal (red arrows, C) on atherosclerotic plaques at 24 hours in LDLR-/- mice injected with LOX-1 antibody probe, but not those injected with nIgG probe (Fig. 1D). The % CNR was significantly higher at 24 hours in LDLR-/- mice that received the probe with LOX-1 antibody compared to nIgG (187.4 ± 71.5% vs. 58.4 ± 46.9%, p < 0.05, Fig 1, bottom left panel). Accordingly, the %NER was also significantly higher at 24 hours in LDLR-/- mice that received the probe with LOX-1 antibody compared to nIgG (121.1 ± 38.9% vs. 18.4 ± 13.9%, p < 0.01, Fig 1, bottom right panel). The atherosclerotic lesions were similar between the 2 groups as determined by H&E staining of aortic cross-sections (G&H).

## Conclusion

MRI with liposomes containing gadolinium and LOX-1 antibody demonstrates specific targeting of atherosclerotic plaques with high contrast to noise ratios. Spiral imaging produced high spatial resolution without motion artifacts and black blood imaging improved visualization of vessel wall. Further study on imaging of LOX-1 may provide more detailed characterization of atherosclerotic plaque *in vivo*. This technique shows significant promise for molecular MR imaging of atherosclerosis.

